# Bicalutamide Enhances Conventional Chemotherapy in In Vitro and In Vivo Assays Using Human and Canine Inflammatory Mammary Cancer Cell Lines

**DOI:** 10.3390/ijms25147923

**Published:** 2024-07-19

**Authors:** Belen Crespo, Juan Carlos Illera, Gema Silvan, Paula Lopez-Plaza, María Herrera de la Muela, Miriam de la Puente Yague, Cristina Diaz del Arco, Paloma Jimena de Andrés, Maria Jose Illera, Sara Caceres

**Affiliations:** 1 Department Animal Physiology, Veterinary Medicine School, Complutense University of Madrid (UCM), 28040 Madrid, Spain; belencre@ucm.es (B.C.); jcillera@ucm.es (J.C.I.); paularlo@ucm.es (P.L.-P.); mjillera@vet.ucm.es (M.J.I.); sacacere@ucm.es (S.C.); 2 Obstetrics and Gynecology Department, Hospital Clinico San Carlos, Instituto de Salud de la Mujer, Instituto de Investigación Sanitaria del Hospital Clínico San Carlos (IsISSC), 28040 Madrid, Spain; maria.herrera@salud.madrid.org; 3 Department of Public and Maternal Child Health University, School of Medicine, Complutense University of Madrid, 28040 Madrid, Spain; mdelapuenteyague@yahoo.es; 4 Department of Surgical Pathology, Hospital Clínico San Carlos, 28040 Madrid, Spain; crisdelarco@gmail.com; 5 Department of Animal Medicine, Surgery and Pathology, Veterinary Medicine School, Complutense University of Madrid, 28040 Madrid, Spain; pjandres@ucm.es

**Keywords:** doxorubicin, docetaxel, bicalutamide, inflammatory mammary cancer, inflammatory breast cancer, steroid hormones, androgen receptor

## Abstract

Human inflammatory breast cancer (IBC) and canine inflammatory mammary cancer (IMC) are highly aggressive neoplastic diseases that share numerous characteristics. In IBC and IMC, chemotherapy produces a limited pathological response and anti-androgen therapies have been of interest for breast cancer treatment. Therefore, the aim was to evaluate the effect of a therapy based on bicalutamide, a non-steroidal anti-androgen, with doxorubicin and docetaxel chemotherapy on cell proliferation, migration, tumor growth, and steroid-hormone secretion. An IMC-TN cell line, IPC-366, and an IBC-TN cell line, SUM149, were used. In vitro assays revealed that SUM149 exhibited greater sensitivity, reducing cell viability and migration with all tested drugs. In contrast, IPC-366 exhibited only significant in vitro reductions with docetaxel as a single agent or in different combinations. Decreased estrogen levels reduced in vitro tumor growth in both IMC and IBC. Curiously, doxorubicin resulted in low efficacy, especially in IMC. In addition, all drugs reduced the tumor volume in IBC and IMC by increasing intratumoral testosterone (T) levels, which have been related with reduced tumor progression. In conclusion, the addition of bicalutamide to doxorubicin and docetaxel combinations may represent a potential treatment for IMC and IBC.

## 1. Introduction

Inflammatory breast cancer (IBC) and inflammatory mammary cancer (IMC) are highly aggressive neoplastic diseases that affect both women and female dogs [1,2]. In humans, IBC is a rare type of breast cancer, with an incidence of 2–2.5% [3]. In female dogs, IMC has an incidence of 18% of all malignant mammary tumors [4,5]. Both IBC and IMC are the most aggressive types of mammary cancer, with worse survival rates than in non-inflammatory cases [1,3,4]. Consequently, research into novel therapeutic approaches for the management of human and canine inflammatory breast cancer has emerged as a significant area of interest in recent years [6]. In addition, IMC shares clinical, biological, pathological, and molecular characteristics with its counterpart disease, IBC [7]. Thus, several studies have refuted that IMC is a great biomodel for the study of IBC [8,9,10].

**Figure 1:**
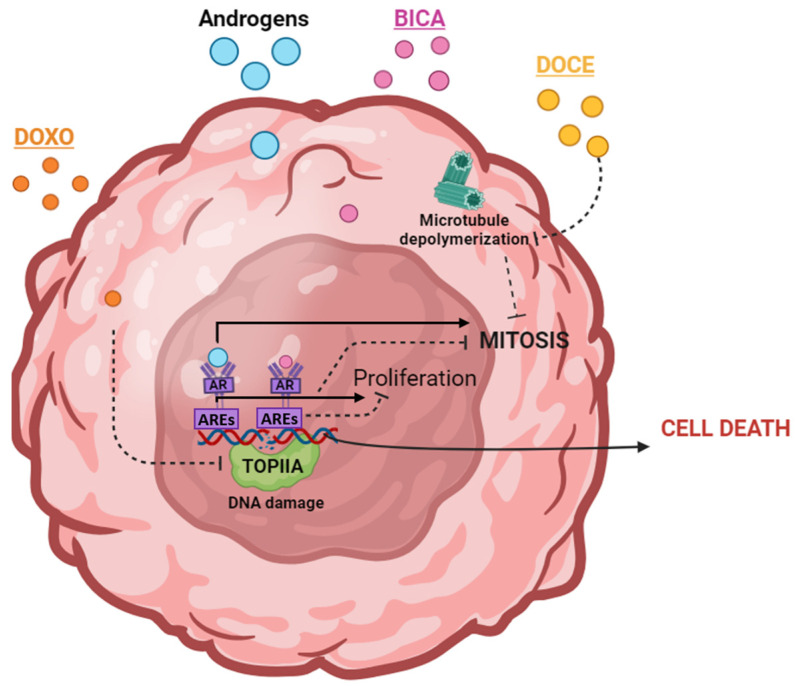
A simple summary of the molecular action mechanisms of doxorubicin (DOXO), docetaxel (DOCE), and bicalutamide (BICA) is represented. Continuous arrows indicate molecular binding or action, while dashed arrows indicate blocking action. The androgen receptor is represented as AR, the androgen response elements as AREs, and topoisomerase II-A as TOPIIA.

In humans, surgery in conjunction with chemotherapy represents the standard treatment for the majority of breast cancer subtypes [11]. In addition to conventional treatment, recent years have seen an increasing focus on personalized therapy based on the specific tumor subtype [11]. This may include anti-hormonal treatments for hormone receptor-positive breast cancers or HER-2-binding monoclonal antibodies for HER-2-overexpressing tumors [12]. This approach has been shown to result in superior disease-free survival compared to surgery and chemotherapy alone [12]. Conversely, surgery represents the first treatment option for canine breast cancer [13]. Chemotherapy, radiotherapy, and hormonal therapy, among others, are also employed for the treatment of canine mammary cancer in some types of tumors [14]. For the IMC subtype, which has a high tendency to metastasize from the early stages of the disease, the application of an effective treatment is complicated to achieve [15]. For this reason, palliative treatment and chemotherapy are selected over surgery in IMC cases [14,16]. However, another additional difficulty is that the criteria for selecting and implementing the type of chemotherapy are not standardized [16]. A few studies have reported the use of doxorubicin, carboplatin, cyclophosphamide, and 5-fluorouracil, among others, with widely scattered results [16]. In some cases of canine mammary cancer, adjuvant chemotherapy has improved the disease-free period [17]. However, in other cases, the benefits have not been as clear [18]. Chemotherapy, both in dogs and in humans, is mainly based on the use of anthracyclines and taxanes [15,19]. One of the most commonly used anthracyclines is doxorubicin, which exerts its effects by binding to DNA-associated enzymes, intercalating between base pairs in the genome, and resulting in DNA damage and the induction of cell apoptosis [20]. Nevertheless, doxorubicin has been associated with significant adverse effects in both humans and dogs, which has led to limitations in the dose that can be supplied [18,21]. On the other hand, docetaxel is a taxane that has been widely used for breast cancer treatment [22]. Docetaxel induces a mitotic block by binding to the beta-tubulin subunit of the microtubules, preventing their disassembly [23] (Figure 1).

On the other hand, approximately 25% of IBC and more than half of diagnosed canine mammary tumors are classified as triple-negative (TNBC) due to the absence of estrogen and progesterone receptor expression and the absence of HER-2 overexpression [24,25]. These tumors are more aggressive and have a worse prognosis [25,26]. In IBC-TN and IMC-TN, neoadjuvant chemotherapy produces a limited pathological response compared to other tumor subtypes, resulting in low survival rates [27,28]. Consequently, research in this field remains of great importance for improving the pathological response in both humans and dogs. One of the factors that has been considered to improve therapeutic response is the study of androgen receptor (AR) expression [29]. The presence of AR has been associated with better disease-free and overall survival [30]. Specifically, it has been clarified that both IMC and IBC exhibit significant endocrine influence and that the use of anti-androgen therapies could potentially reduce tumor progression [31,32]. Endocrine therapies are currently under investigation for the influence of steroid hormones on tumor growth in human and canine mammary cancers [33,34]. Androgens are derived from cholesterol, which can be converted to dihydroepiandrostenedione (DHEA) and androstenedione (A4) [26,35]. In addition, A4 can be converted to testosterone (T) or estrone (E1), resulting in the formation of dihydrotestosterone (DHT) or 17β-estradiol (E2), respectively [26,36,37]. In recent years, there has been a growing interest in the potential of anti-androgen therapies for the treatment of breast cancer [38]. Treatment options whose target is the AR, such as bicalutamide, a second-generation non-steroidal AR antagonist, have been widely used in prostate cancer with successful results [39,40]. Currently, these treatments are under investigation in the context of breast cancer, with preliminary results indicating that they may also have clinical benefit rates for this human disease [19]. It has been observed that the use of anti-androgenic treatments in combination with conventional chemotherapy could improve outcomes in patients with breast cancer [41].

Thus, the addition of an anti-AR treatment could enhance the efficacy of conventional chemotherapy based on anthracyclines and taxanes in IBC-TN and IMC-TN. The aim of this study was to evaluate the effect of adding bicalutamide to conventional chemotherapy based on doxorubicin and docetaxel on the study of proliferative, migratory, and tumor-growth processes, as well as on steroid-hormone secretion.

## 2. Results

### 2.1. Sensitivity Assay

**Figure 2:**
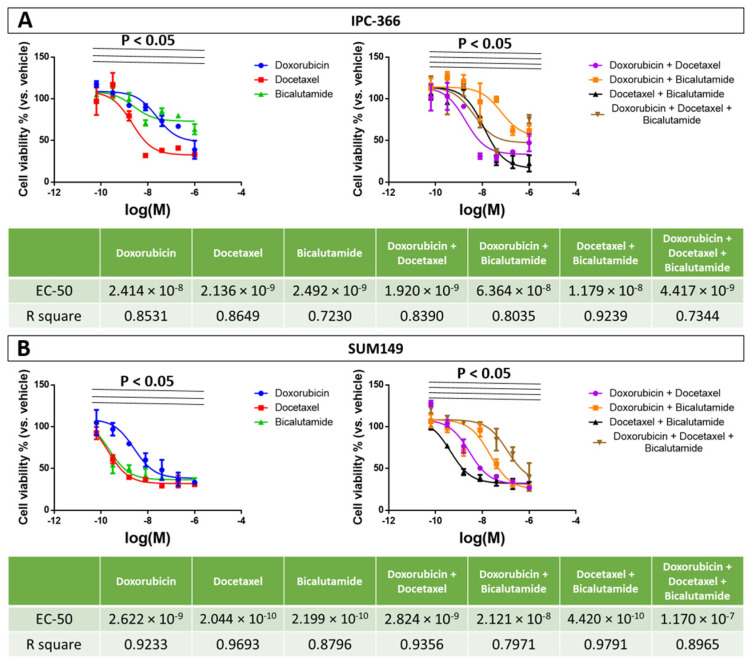
Sensitivity assay results of doxorubicin, docetaxel, bicalutamide, and their combinations in IPC-366 cell line (**A**) and SUM149 cell line (**B**). Results are expressed graphically with cell viability percentage as a function of the molarity logarithm (Log (M)) of each drug. EC-50 values and R-square of each drug are summarized in the tables below. *p* < 0.05 denotes significant differences between doses.

The sensitivity assay was performed with single treatments (doxorubicin, docetaxel, and bicalutamide) and with different combinations (doxorubicin plus docetaxel, doxorubicin plus bicalutamide, docetaxel bicalutamide, and doxorubicin plus docetaxel plus bicalutamide). The results obtained are summarized in Figure 2. SUM149 cells exhibited slightly greater sensitivity to treatments than IPC-366. However, doxorubicin treatment appeared to be less sensitive in both cell lines. Regarding treatment combinations, the triple combination (doxorubicin plus docetaxel plus bicalutamide) was the most sensitive for IPC-366 cells; while the application of docetaxel and bicalutamide, as a single treatment or their combination, was more sensitive in SUM149 cells. On the contrary, the triple combination in SUM149 cells was the least-sensitive treatment combination.

### 2.2. Immunofluorescence

**Figure 3:**
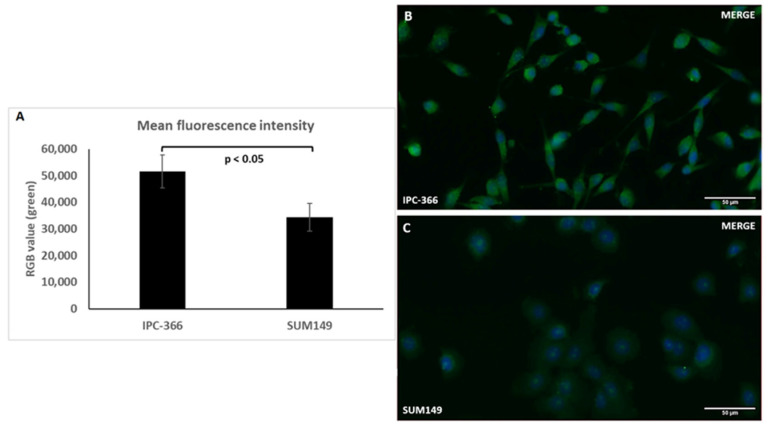
Images were taken at 40× magnification at 50 µM scale. Data from the quantification of AR immunofluorescence is expressed graphically as AR expression mean ± standard deviation (SD) (**A**). Immunofluorescence results confirm the AR expression (green) in IPC-366 (**B**) and SUM149 (**C**) cell lines. Cells were DAPI-stained to visualize cell nuclei (blue).

SUM149 and IPC-366 cells showed AR expression by immunofluorescence but differed in the intensity of the expression. After performing fluorescence quantification with ImageJ software 1.53e version, the results confirmed significant differences (*p* < 0.001) in AR expression between IPC-366 and SUM149, with higher expression in IPC-366 (Figure 3A). Cytoplasmic and nuclear expression could be observed in IPC-366 (Figure 3B); however, in SUM149, the expression, although very light, is mostly found in the nucleus (Figure 3C).

### 2.3. Cell Viability Assay

**Figure 4:**
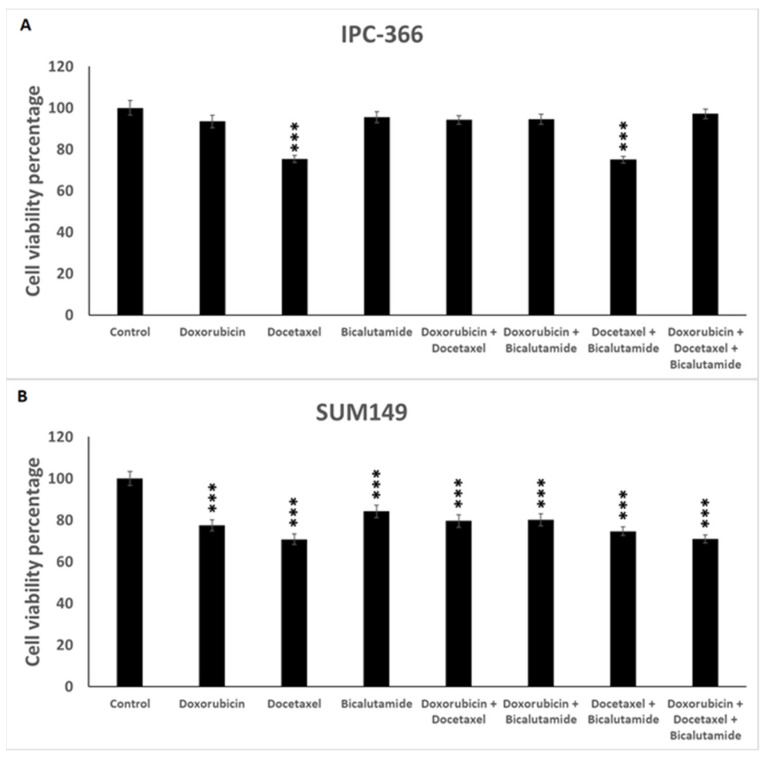
Cell viability assay of IPC-366 (**A**) and SUM149 (**B**) cultured cells. Bars represent the cell viability reduction produced by doxorubicin, docetaxel, bicalutamide, and their combinations. *** Denoted *p* < 0.001 significant differences between control and treatments.

These results demonstrate that SUM149 exhibited greater reductions in cell viability than IPC-366. IPC-366 obtained significant reductions (*p* < 0.001) with docetaxel as a single treatment and a docetaxel-plus-bicalutamide combination. In contrast, doxorubicin alone or in combination with docetaxel and/or bicalutamide reduced IPC-366 cell viability by only about 5% with respect to the control, with the doxorubicin being less effective than other drugs (Figure 4A). On the other hand, SUM149 reduced cell proliferation significantly (*p* < 0.001) with all treatments (Figure 4B). The application of docetaxel alone and the triple combination reduced cell viability in SUM149 to 70%. Similarly, in IPC-366, the application of docetaxel and the combination of docetaxel with bicalutamide reduced cell viability to 75%. Therefore, docetaxel appears to play a significant role in reducing cell viability in IMC and IBC cells. Conversely, the drugs that exhibited a poor reduction in cell viability were bicalutamide in SUM149 and the triple combination in IPC-366, with cell viability reduced to 84% and 97%, respectively.

### 2.4. Analysis of Drug Interactions

**Figure 5:**
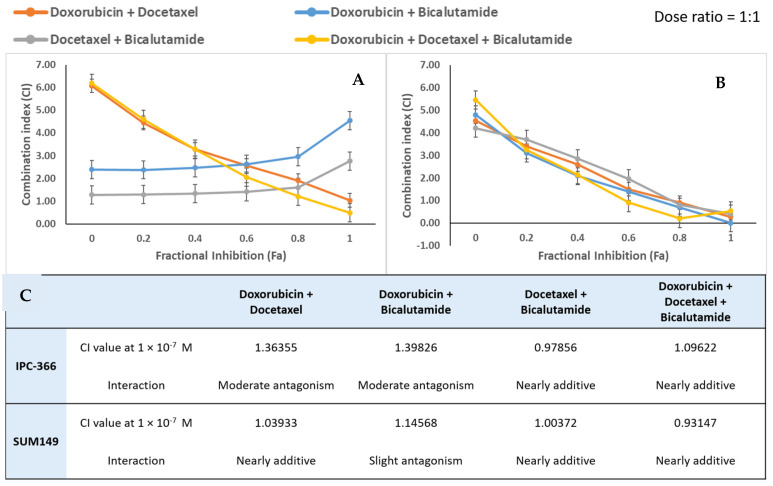
Combination index (CI) versus fractional effect (Fa) curve as described by Chou and Talalay model [41] on IPC-366 (**A**) and SUM149 (**B**) cells exposed to doxorubicin plus docetaxel, doxorubicin plus bicalutamide, docetaxel plus bicalutamide, and doxorubicin plus docetaxel plus bicalutamide (1:1). Each point represents the CI ± SD at a fractional effect as determined in our experiments. The line (CI = 1) indicates additivity, the area under this line, synergism, and the area above the line, antagonism. The table represents the CI value at the dose used in in vitro assays and the pharmacological interaction described by Chou and Talalay [42] (**C**).

The Chou-Talalay method was used to calculate the combination index (CI) using CompuSyn software 2004 version. Drug interactions (antagonist, additive, or synergic) were classified as strong, moderate, or slight, depending on the range of the CI (Figure 5A,B). Doxorubicin and docetaxel combination obtained a CI of 1.36 in IPC-366 and a CI of 1.03 in SUM149 (Figure 5C), which corresponded to moderate antagonism and nearly additive, respectively. However, the combination of docetaxel and bicalutamide, as well as the triple combination, demonstrated a nearly additive interaction in both cell lines. Finally, the doxorubicin and bicalutamide combination exhibited a slight antagonism in SUM149 and a moderate antagonism in IPC-366.

### 2.5. Wound Healing Assay

**Figure 6:**
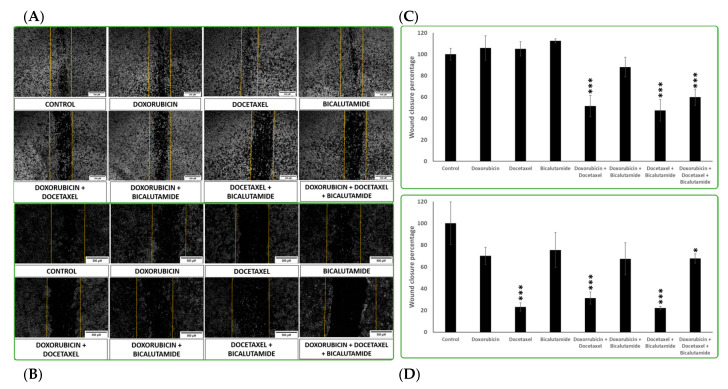
Images from the wound-healing assay were recorded 24h after performing the wound. Representative images from the control and the different treatments, IPC-366 (**A**) and SUM149 (**B**), are shown, as well as graphs that represent the wound-closure percentage in IPC-366 (**C**) and SUM149 (**D**). Images were taken at 10x magnification and processed with ImageJ software Wound Healing Tool 1.53e version with a scale bar of 500 µm. Bars represent the mean percentage wound closure ± SD. * Denoted *p* < 0.05, and *** *p* < 0.001 significant differences.

The addition of treatments in both cell lines resulted in differences in migration capacity, especially when combinations of treatments were administered (Figure 6A,B). In general, both cell lines reduced their cell migratory capacity with the combination of treatments more than when the treatments were applied as a single agent, with the exception of docetaxel in SUM149, which migrated 76.85% less than the control after 24 h (Figure 6D). The most effective treatment for reducing cell migration was docetaxel combined with bicalutamide in both IPC-366 and SUM149, which migrated 52.47% and 83.37% less than the control, respectively (Figure 6C,D). In contrast, bicalutamide was the least effective treatment in decreasing cell migration, especially in IPC-366, where it closed 10% of the wound more than the control.

### 2.6. Tumor Growth

**Figure 7:**
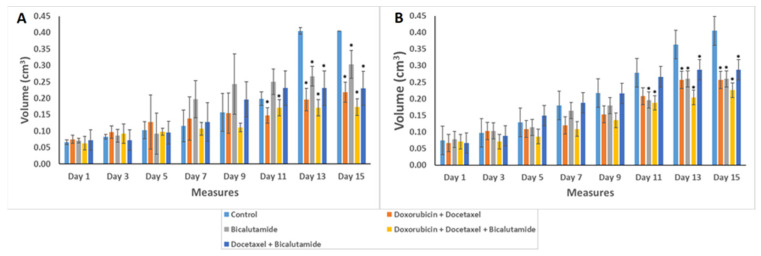
Graphs represent tumor volume in SCID mice inoculated with IPC-366 (**A**) or SUM149 (**B**) cells. Animals were treated with 2.5 mg/kg/day of doxorubicin, 8 mg/kg/day of docetaxel injected once per week intraperitoneally, or 10 mg/kg/day of bicalutamide added in the drinking water for 15 days. Bar represents the means of tumor volume ± SD. * Denoted *p* < 0.05, significant differences between control and treatments.

Animals inoculated with IPC-366 and SUM149 cells were subsequently treated with different drugs (bicalutamide; doxorubicin and docetaxel; docetaxel and bicalutamide; and doxorubicin, docetaxel, and bicalutamide). Results revealed that all treatments administered in IPC-366 and SUM149 mice showed a reduction in tumor growth (Figure 7A,B). From day 11 of treatment onwards, the combination of doxorubicin and docetaxel and the triple combination presented significantly smaller tumor volumes than the control in both cell lines. In SUM149, the bicalutamide group and the docetaxel plus bicalutamide group also resulted in a significant reduction of tumor volume with respect to the control group. However, IPC-366 mice did not show that reduction in those groups of treatment until day 13. Interestingly, the triple combination obtained the most favorable outcomes in both canine and human xenotransplanted mice. On the other hand, bicalutamide and docetaxel plus bicalutamide were the treatments that demonstrated the least efficacy in reducing tumor volume in IPC-366 and SUM149 mice, respectively.

### 2.7. Hormone Concentrations in Culture Media, Serum, and Tumor Homogenates

**Figure 8:**
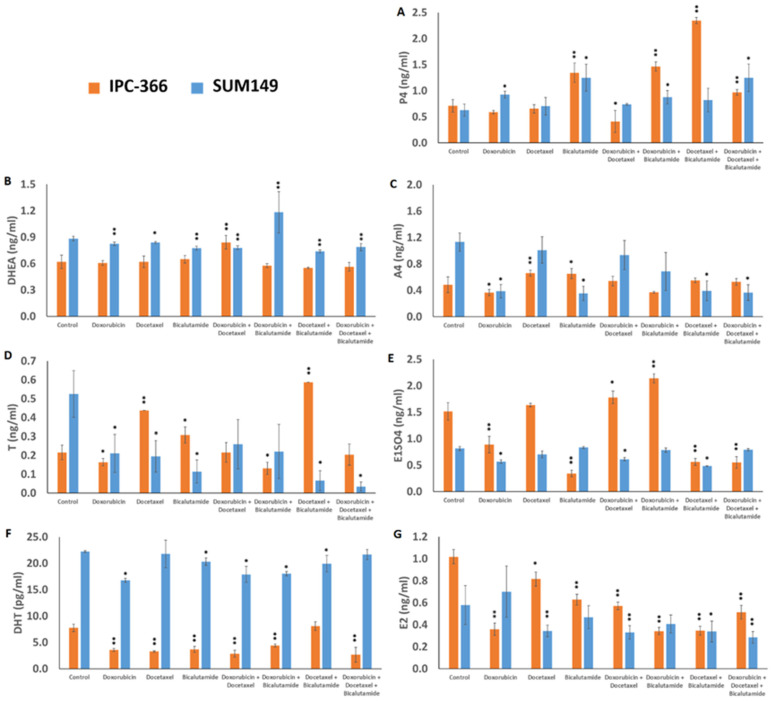
Graphs represent hormone concentrations obtained from culture media with each single treatment and their combinations. Bars represent means ± SD. (**A**) P4, (**B**) DHEA, (**C**) A4, (**D**) T, (**E**) E1SO4, (**F**) DHT, and (**G**) E2 levels were determined. * Denoted *p* < 0.05, and ** *p* < 0.01 significant differences between control and treatments.

All treatments produced a steroid-hormone alteration with respect to the control, either in in vitro or in in vivo assays. Culture media hormonal results revealed that both cell lines had different hormonal concentrations as the SUM149 control group showed higher androgen levels (DHEA, A4, T, DHT) than the IPC-366 control group, which revealed higher estrogen levels (E1SO4 and E2) (Figure 8). In vitro studies have demonstrated that SUM149 will use androgen levels to maintain estrogen levels similar to control and promote cell survival. Furthermore, it has been observed that a reduction in androgen and E1SO4 levels is associated with a significant decrease in cell viability and migration of SUM149. Conversely, IPC-366 has been shown to significantly reduce cell viability and migration when DHT levels decrease. The hormonal fluctuations observed in vitro are detailed below.

Regarding IPC-366, P4 levels showed a significant increase (*p* < 0.01) in bicalutamide treatment and its combinations with doxorubicin, docetaxel, and the triple combination. However, the doxorubicin and docetaxel combination significantly reduced (*p* < 0.05) P4 levels in IPC-366. Additionally, SUM149 showed a significant increase (*p* < 0.05) in P4 levels with doxorubicin and bicalutamide treatments as single agents, in combination, and with the triple combination (Figure 8A). Regarding DHEA levels, no significant differences were found in IPC-366 treatments, except with the doxorubicin and docetaxel combination, which increased DHEA levels significantly (*p* < 0.01). SUM149 resulted in a significant decrease (*p* < 0.01) of DHEA levels in all treatments except for doxorubicin plus bicalutamide, where DHEA levels were significantly higher (*p* < 0.01) (Figure 8B). While androgens such as A4 and T exerted hormonal variations depending on treatment conditions (Figure 8C,D), other androgens such as DHT levels were reduced under all treatment conditions and both cell lines (Figure 8F). With respect to A4 and T levels, it was interesting to observe that both hormones showed similar patterns in both cell lines under all treatment conditions except for the docetaxel plus bicalutamide treatment, where T levels showed a significant increase (*p* < 0.01) in IPC-366 cells, while in SUM149, A4 and T levels showed a significant decrease (*p* < 0.05). In general, all treatments exerted a reduction in A4 and T levels in SUM149 cells, being significant (*p* < 0.05) in treatments with doxorubicin and bicalutamide as single agents and with the triple combination. In contrast, IPC-366 decreased (*p* < 0.05) A4 and T levels in doxorubicin as a single treatment or in combination with bicalutamide only in T levels. In contrast, estrogens were decreased under almost all treatments in IPC-366. E2 levels were decreased with all treatments, but E1SO4 levels increased with the doxorubicin plus docetaxel and doxorubicin plus bicalutamide combinations (Figure 8E,G). In SUM149, E2 levels decreased significantly (*p* < 0.01) with docetaxel and its combination with doxorubicin and bicalutamide and the triple combination (Figure 8G).

**Figure 9:**
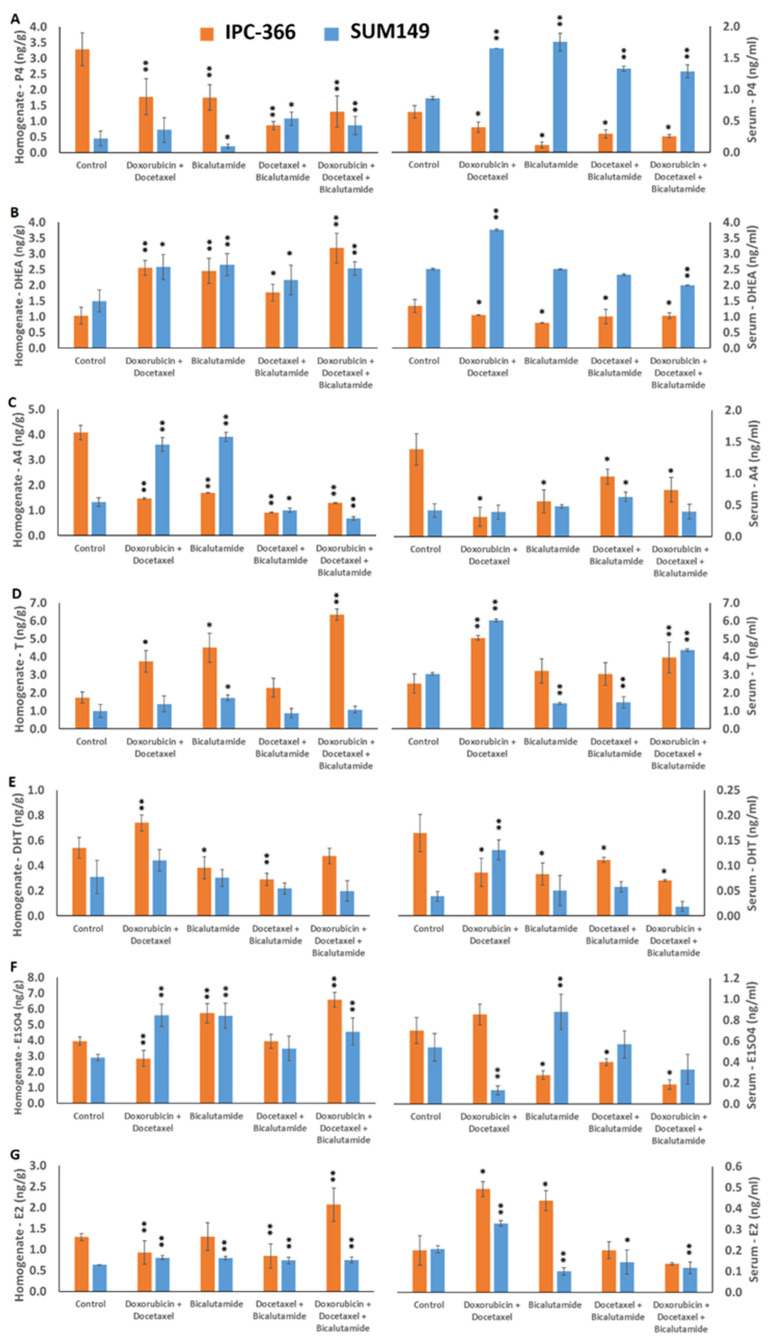
Graphs represent the hormone concentration obtained from serum samples (right panel) and tumor homogenate (left panel) with each single treatment and their combinations. Bars represent means ± SD. (**A**) P4, (**B**) DHEA, (**C**) A4, (**D**) T, (**E**) DHT, (**F**) E1SO4, and (**G**) E2 levels were determined. * Denoted *p* < 0.05, and ** *p* < 0.01 significant differences between control and treatments.

In vivo, elevated T levels of both IPC-366 and SUM149 in tumor homogenate and serum were associated with a reduction in tumor expansion. Furthermore, an increase in E1SO4 levels in the tumor homogenate was associated with a mechanism of cell survival in both IMC and IBC. A more detailed examination of hormone levels reveals the following: Regarding serum and tumor-homogenate hormone concentrations in Balb/SICD mice inoculated with IPC-366 and SUM149 cells, alterations in hormonal homeostasis were found when mice were administered with the different treatment combinations. IPC-366-treated mice presented a significant decrease in P4 serum and tumor-homogenate levels (*p* < 0.05) with respect to the control, while in SUM149 mice, an increase in serum tumor homogenate levels in all treatment groups was found (Figure 9A). Indeed, treatment combinations produced a significant increase in tumor-homogenate levels (*p* < 0.05) of IPC-366 and SUM149 with all treatments. However, serum DHEA levels in IPC-366 mice significantly decreased (*p* < 0.05) with all treatments, and in SUM149 mice, these levels tended to increase significantly with the administration of doxorubicin plus docetaxel and with the triple combination (Figure 9B). A4 tumor homogenates and serum levels showed a significant decrease (*p* < 0.05) in IPC-366 mice with all treatments, while SUM149 mice revealed a significant increase (*p* < 0.05) with the combination of doxorubicin and docetaxel and with bicalutamide as a single agent (Figure 9C). All the treatments appeared to increase T-tumor homogenate and serum levels in IPC-366 and SUM149 mice, except for SUM149 serum T levels, which significantly decreased (*p* < 0.05) with bicalutamide and docetaxel plus bicalutamide treatments (Figure 9D). Interestingly, the DHT serum tumor homogenate of SUM149 mice did not show any significant alteration, though serum and tumor-homogenate DHT levels of IPC-366 mice significantly decreased (*p* < 0.05) except for with the doxorubicin plus docetaxel treatment, in which tumor-homogenate DHT levels significantly increased (*p* < 0.05) (Figure 9E).

Serum and tumor-homogenate estrogen levels were also altered with the administration of the different treatments. E1SO4 and E2 serum and tumor-homogenate levels in SUM149 mice showed similar results: while in tumor homogenates, estrogen levels significantly increased (*p* < 0.05) with treatment, and serum estrogen levels decreased. However, in IPC-366 mice, E1SO4 levels in tumor homogenates increased with treatments, and E2 decreased with respect to the control group. Similarly, serum E1SO4 and E2 levels followed the same pattern: E1 serum levels decreased with the administration of treatments while E2 serum levels increased (Figure 9F,G).

## 3. Discussion

Conventional chemotherapy produces a limited pathological response in IMC and IBC [15,18,19,28]. In light of these limitations, targeted therapies have emerged as a promising avenue for improving therapeutic response in recent years [29]. Some researchers have demonstrated that anti-hormonal treatments in conjunction with chemotherapy could enhance outcomes in TNBC patients [41,43], reducing disease progression more effectively than conventional chemotherapy and surgery alone [31,32,33,44,45]. Both breast and prostate cancers are highly hormone-dependent diseases, and, as a result, many of the therapies used for prostate cancer are being investigated for breast cancer [46]. Steroid hormones are associated with tumor development in both cases, and several studies have refuted the hypothesis that endocrine therapies could be beneficial for those carcinomas [32,46]. Besides, the advantages of anti-hormonal treatments such as bicalutamide include a reduction in adverse effects that may be well tolerated by patients, such as hot flashes, nausea, and mild malaise [47]. In this study, the efficacy of bicalutamide addition, a second-generation AR antagonist that is widely used in prostate cancer, to conventional chemotherapy (doxorubicin and docetaxel) used in breast cancer has been evaluated for IMC and IBC [15,19,39,40].

Comparative studies between canine mammary cancer and human breast cancer have determined that canine mammary cancers are suitable animal models for comparing the prognosis and treatment results between the two species [33,34,48]. Caceres et al. (2015) established the first IMC-TN cell line, IPC-366, which has been used for numerous research studies [49]. IPC-366 has been demonstrated to be a good model comparison with its human counterpart, SUM149, due to the similarity of patterns observed in both cell lines [9,10]. This study also presented evidence of the similarities between IMC and IBC, as IPC-366 and SUM149 presented a similar in vitro and in vivo response to the treatments used. Previous studies have examined the AR expression in both IMC and IBC [31,32]. Nevertheless, a comparison of AR-expression intensity between the two cell lines has not been made. Immunofluorescence revealed higher AR expression in IPC-366, suggesting that treatments that interfere with AR functionality, such as bicalutamide, could trigger different effects in the two cell lines.

In this study, both cell lines proved to be sensitive to doxorubicin, docetaxel, and bicalutamide, as well as their combinations. However, the IBC-TN cell line, SUM149, exhibited greater sensitivity in vitro, as evidenced by a reduction in cell viability and migration under all tested drugs. In contrast, the IMC-TN cell line, IPC-366, only significantly reduced its cell viability and migratory capacity with the administration of docetaxel as a single agent or with the different combinations.

It is noteworthy that doxorubicin treatment showed limited efficacy when it was administered independently, particularly in IPC-366. It has been proposed that doxorubicin-based chemotherapy did not result in an improved outcome in female dogs with advanced mammary cancer compared to surgery alone [18]. Anthracyclines have as their main target the topoisomerase II-alpha (TOP2A) gene, which is located in the same amplicon as the *HER2/neu* gene [50]. In TNBC cases, there is no overexpression of HER-2, and doxorubicin has been found to be less effective [50,51].

Curiously, doxorubicin appears to affect the enzymatic activity of the 3β-hydroxysteroid dehydrogenase (3βHSD) and 17β-hydroxysteroid dehydrogenase (17βHSD), altering steroid-hormone production [52]. The enzyme 3βHSD converts DHEA to A4, and 17βHSD converts T from A4, denoting that doxorubicin could interfere with androgen synthesis [53]. Our results revealed that in vitro, doxorubicin treatment caused a decrease in A4 and T levels in both cell lines, so doxorubicin was altering the enzymatic activity of 3βHSD and 17βHSD in IPC-366 and SUM149. However, only cell viability in SUM149 was affected under doxorubicin; neither cell viability in IPC-366 nor migration in both cell lines was significantly decreased under doxorubicin as a single agent. On the other hand, docetaxel is a taxane that disrupts microtubule function, so its use could be beneficial in limiting tumor growth by preventing tumor cell division [54]. Docetaxel as a single agent reduced cell viability in IPC-366 and SUM149 and cell migration in SUM149. This is consistent with sensitivity results, which showed that SUM149 was more sensitive to docetaxel application than IPC-366. In terms of hormonal secretion, T levels in IPC-366 increased with docetaxel treatment and decreased with doxorubicin. The differences in AR expression between IPC-366 and SUM149 could suggest different susceptibility to therapies that interfere with AR function, presenting a different hormonal tendency. IPC-366, with high AR expression, could be more susceptible to androgenic variations. Thus, an increase in T levels could lead to a decrease in cell viability in IPC-366. In contrast, E2 levels in SUM149 decreased with docetaxel, and E1SO4 levels decreased with doxorubicin. Therefore, a decrease in SUM149 estrogen levels could be associated with decreased progression of breast cancer [10,34]. For these reasons, it is evident that the limited efficacy of doxorubicin in female dogs and the severe adverse effects it produces, including cell toxicity and congestive heart failure, indicate that the avoidance of doxorubicin could enhance the quality of life for patients [21].

On the other hand, the doxorubicin and docetaxel combination produced a better response than when doxorubicin was administered as a single agent in terms of cell viability and cell migration in both cell lines. The pharmacological interaction study revealed that the combination of doxorubicin and docetaxel in SUM149 has an additive effect, according to Chou and Talalay’s study [42]. The additive effect is defined as the overall effect of combined drugs being the sum of the pharmacological effects of each individual agent [55]. In contrast, in IPC-366, combined chemotherapy has a slight antagonism effect, which is defined as a reduction in the effect of combined agents compared to the effect of individual drugs [55]. Consequently, cell viability was reduced by doxorubicin plus docetaxel in SUM149, but it was not affected in IPC-366. E1SO4 levels in IPC-366 were increased. It is well known that elevated E1SO4 levels are associated with an increase in tumor volume, serving as a reservoir for IMC-TN to avoid cell viability reduction [10], contrary to SUM149, where E1SO4 levels are reduced, and cell viability is compromised. Curiously, combined chemotherapy reduced cell migration significantly in both cell lines. P4 and DHT levels were reduced in IPC-366. High levels of progesterone have been related to breast carcinogenesis [56]. Consequently, a decrease in P4 levels could be associated with a decrease in cell migration. With respect to androgen levels, the DHT levels with doxorubicin plus docetaxel and with the triple combination were the lowest compared to the other treatments, so low DHT levels could be related to a significant decrease in cell migration. This is in agreement with other authors who state that androgens are related to cell metastasis capacity due to AR promoting epithelial-mesenchymal transition [45].

The influence of steroid hormones and AR’s role on breast cancer tumor development is a target that is currently being addressed for the improvement of current therapies [29]. Some researchers have linked anti-androgen therapies to a benefit in patients with metastatic triple-negative AR-positive metastatic breast cancer [57]. Bicalutamide was applied as a single agent and in combination with the different chemotherapeutics to assess its potential to enhance the efficacy of chemotherapy. The application of bicalutamide produced significant reductions in SUM149 proliferation in vitro. The AR antagonist produced in SUM149 a decrease in androgen-secreted levels, maintaining estrogen levels similar to the control. Therefore, neoplastic cells with low AR expression could be using androgens for estrogen formation in order to survive when cell viability is compromised. In IPC-366 with high AR expression, cell proliferation was not diminished with the application of bicalutamide as a single agent. Osguthorpe and Hagler (2011) observed that bicalutamide could exert a slight partial agonist function in prostate cancer due to its binding site of the AR [58]. This effect may also occur in the IMC cell line with high AR expression, making bicalutamide less effective in this cell line.

On the other hand, the combination of docetaxel with bicalutamide reduced both proliferation and migration in IPC-366 and SUM149. After having analyzed the pharmacological interaction between docetaxel and bicalutamide, the nearly additive effect of the combined drugs confirms that the cell viability reduction in both cell lines is mostly due to docetaxel, since the reduction produced by docetaxel alone is very similar to that produced by the combination of both treatments. However, the additive effect of the combination treatment on IPC-366 produced a greater reduction in cell migration than both treatments as single agents. At hormonal levels, the presence of docetaxel plus bicalutamide in vitro increased T secretion in IPC-366 more than with both drugs separately, so bicalutamide appears to avoid secreted androgens consumption, which, together with docetaxel action, reduces cell viability and migration. On the other hand, androgen levels in SUM149 with the presence of the combined treatment were reduced, leading to a decrease in cell migration. Giovannelli et al. (2019) suggested that androgens promote the formation of the AR/Src complex, which leads to TNBC cell migration and invasiveness [44]. In addition, Rajarajan et al. (2023) have linked AR expression with increased epithelial-mesenchymal induction, observing that the use of anti-androgen therapies reduced cell migration, which is consistent with our study [59].

In contrast, the doxorubicin and bicalutamide combination only reduces cell viability in SUM149. Combined treatments exert a moderate and slight antagonism in IPC-366 and SUM149, respectively. Elevated levels of E1SO4 found in IPC-366 could be maintaining cell viability in vitro [10]. Therefore, the doxorubicin and bicalutamide combination was the least effective in treating IMC and IBC, and the use of docetaxel in combination with bicalutamide achieved the best in vitro results in both cell lines.

Therefore, the in vitro results clarified that the administration of docetaxel as a single agent obtained good results in terms of the reduction in cell proliferation and migration in both cell lines. However, the addition of bicalutamide to docetaxel obtained better results than when docetaxel was administered alone or in combination with doxorubicin, denoting that the addition of bicalutamide could enhance docetaxel efficiency.

Nevertheless, the combination of the three drugs in vitro exerts an additive effect, according to Chou and Talalay’s study [42]. The doxorubicin plus docetaxel plus bicalutamide produces a reduction in androgen levels in both cell lines, reducing cell migration. In SUM149, the levels of A4 and T are decreased, which could be due to the addition of doxorubicin and its interaction with the enzymes 3βHSD and 17βHSD, being more effective in SUM149 and reducing cell viability.

In vivo studies showed that doxorubicin, docetaxel, and bicalutamide had great efficacy in reducing tumor volume. The triple combination obtained the greatest reductions in IPC-366 and SUM149 mice, denoting that the administration of bicalutamide enhances the efficacy of conventional chemotherapy in IMC and IBC tumors. Therefore, the addition of anti-hormonal treatments to conventional treatment regimens is of great utility in treating breast cancer. In IMC-TN, doxorubicin plus docetaxel produced a similar reduction in tumor volume as docetaxel with bicalutamide. Therefore, as doxorubicin exerted limitations, the use of docetaxel in the treatment of female dogs could be a good treatment option due to its in vivo efficacy and good tolerability, as confirmed by other authors [13,18].

Reduced tumor volume with the addition of bicalutamide plus doxorubicin plus docetaxel treatment could be partially explained by the hormonal alterations. There is strong evidence that neoplastic mammary cancer cells can produce steroids from circulating precursors and that treatments alter hormonal homeostasis, promoting or reducing tumor expansion [34,36]. In the triple combination treatment, an increase in serum and tumor-homogenate T levels was found in IPC-366 and SUM149 xenotransplanted mice, which is in line with other studies [33,34]. Therefore, these results support the hypothesis that elevated serum and tumor-homogenate T levels inhibit tumor growth in IMC and IBC. In addition, E1SO4 levels in the tumor homogenate were found to be elevated with the triple combination in both IPC-366 and SUM149 mice. The number of estrogens found intratumorally may act as estrogen reservoir [10,36,60] that may be involved in the mechanism of cell survival [1,10,33].

This study aims to investigate inflammatory TNBC, which has a higher relapse and mortality rate. These preliminary results demonstrate benefits in the future for both canine and human clinical practice. Furthermore, the study of the pharmacological interaction of the drug combination may predict its in vivo efficacy. The strength of this study lies not only in its coverage of both human and canine species but also in its emphasis on the impact of adding an AR antagonist treatment to conventional chemotherapy and its influence on steroid-hormone synthesis. On the other hand, the moderate antagonism effect of doxorubicin and docetaxel in IPC-366 could be the explanation for why some authors have found the application of combination chemotherapy ineffective in female dogs with IMC. It is worth highlighting the importance of anti-androgen therapies for inflammatory mammary cancer in women and female dogs. This study confirms that the addition of an anti-AR drug increases chemotherapy’s efficacy. In addition, it has been observed that the use of docetaxel in IBC, and especially in IMC, is more effective than the use of doxorubicin.

## 4. Materials and Methods

### 4.1. Inflammatory Mammary Cancer Cell Lines

IPC-366, the canine inflammatory triple-negative mammary cancer (IMC-TN) cell line, was obtained from the Department of Physiology, School of Veterinary Medicine (University Complutense of Madrid, Spain). The cells were cultured in Dulbecco’s modified Eagle medium nutrient mixture F-12 Ham (DMEM/F12) (Sigma-Aldrich, St. Louis, MO, USA), supplemented with 5% fetal bovine serum (FBS), 1% penicillin-streptomycin solution, and 1% L-glutamine (Sigma-Aldrich, St. Louis, MO, USA).

SUM149, the human inflammatory triple-negative breast cancer (IBC-TN) cell line, was purchased from Asterand, Plc. (Detroit, MI, USA). It was cultured in Nutrient Mixture F-12 HAM medium (Sigma-Aldrich, St. Louis, MO, USA) supplemented with 5% FBS, 1 µg/mL hydrocortisone, 5 µg/mL insulin, and 1% penicillin-streptomycin solution (Sigma-Aldrich, St. Louis, MO, USA).

Both cell lines were cultured in 25-cm^2^ culture flasks and maintained at 37 °C in a humidified 5% carbon dioxide atmosphere. Cell cultures were monitored daily by phase-contrast microscope (Optika XDS-2 Inverted Microscope, Euromicroscopes, S.L., Barcelona, Spain) to determine cell viability and growth.

### 4.2. Treatments

Doxorubicin hydrochloride (D1515–10MG), docetaxel (01885-5MG–F), and bicalutamide (B9061-10MG) were purchased from Sigma-Aldrich (St. Louis, MO, USA). The drugs were diluted in dimethyl sulfoxide (DMSO) (Sigma Aldrich, St. Louis, MO, USA) and stored at 4 °C until use.

For in vitro assays, single drugs doxorubicin, docetaxel, and bicalutamide, as well as combination treatments, were used at a concentration of 100nM as follows: doxorubicin plus docetaxel, doxorubicin plus bicalutamide, docetaxel plus bicalutamide, and doxorubicin plus docetaxel plus bicalutamide. In vivo assays were performed based on the outcomes obtained in the in vitro assays. The groups used in the in vivo assay were as follows: bicalutamide; doxorubicin and docetaxel; docetaxel plus bicalutamide; and the triple combination (doxorubicin, docetaxel, and bicalutamide). The dosage of the different compounds was selected based on previous studies [43,51,61]: 2.5 mg/kg of doxorubicin and 8 mg/kg of docetaxel were injected intraperitoneally once per week, with a three-day interval between each compound for 15 days. Bicalutamide was administered in the drinking water at a dosage of 10 mg/kg/day for the 15 days.

### 4.3. Sensitivity Assay

The sensitivity assay was performed to determine the sensitivity of IPC-366 and SUM149 to different dilutions of each compound [31]. The assay was carried out in duplicate. IPC-366 and SUM149 cell lines were seeded at a density of 10^3^ cells in 96-well polystyrene plates (Corning Incorporated, New York, NY, USA). Subsequently, 5-fold serial dilutions were performed, starting from a dose of 10mM, in order to achieve a concentration of 64 nM of each drug. All compounds were diluted in DMSO, and a control (cells only with DMSO) was performed to consider the possible toxicity generated by the DMSO. Cells were incubated for 72 h in a humidified 5%-carbon-dioxide atmosphere at 37 °C. Finally, bromide of 3-(4,5-dimetiltiazol-2-ilo)-2,5-difeniltetrazol (MTT) (Sigma-Aldrich, St. Louis, MO, USA) was added to all the wells. The absorbance was measured at a wavelength of 568 nm with an automated plate reader (ThermoFisher Scientific, Waltham, MA, USA). The results were processed with GraphPad Prism 6.01 software, and an EC-50 value was obtained for each drug and cell line.

### 4.4. Immunofluorescence

For the study of the expression of AR in IPC-366 and SUM149 cell lines, 5 × 10^4^ cells were plated on culture chambers (Sarstedt, Germany). Then, the cultured cells were fixed with 4% paraformaldehyde (Invitrogen, Waltham, MA, USA) and permeabilized with 0.5% Triton X-100 (Sigma-Aldrich, St. Louis, MO, USA). The cells were blocked with goat serum and incubated with a rabbit primary AR-polyclonal antibody (PA1-37079) (Sigma-Aldrich, St. Louis, MO, USA) overnight at 4 °C. After, cells were washed with PBS and incubated with a secondary antibody (CF488A; Sigma Aldrich, St. Louis, MO, USA). Finally, the slides were mounted with Prolong Gold Antifade with DAPI (Invitrogen, MA, USA). Images were captured using an Optika fluorescence microscope (Optika IM-3LD2 Microscope, Bérgamo, Italy). A total of one hundred cells were evaluated in three different fields, with those that emitted fluorescence designated as positive. Additionally, immunofluorescence quantification was analyzed with the RGB value plugin available in the ImageJ software 1.53e version in order to compare the intensity of expression between IPC-366 and SUM149.

### 4.5. Cell Viability Assay

Both cell lines, IPC-366 and SUM149, were seeded in 96-well polystyrene plates (Corning Incorporated, New York, NY, USA) at a density of 10^4^ cells per well in a culture medium containing 100 nM of doxorubicin, docetaxel, bicalutamide, and their combinations.

Cells were cultured during 24 h at 37 °C in a humidified atmosphere containing 5% CO_2_. The assay was carried out in duplicate, with untreated cells serving as a control. Finally, MTT was added, and the absorbance was measured at a wavelength of 568 nm with an automated plate reader (ThermoFisher Scientific, Waltham, MA, USA). The data were expressed as a percentage of cell viability with respect to the control cells.

### 4.6. Analysis of Drug Interactions

IPC-366 and SUM149 were seeded in 96-well polystyrene plates (Corning Incorporated, New York, NY, USA) at a density of 10^4^ cells per well. The assay was carried out in duplicate. A series of drug combinations were prepared in a fixed ratio of 1:1, with doses that corresponded to 0.5, 1, and 1.5 times that of the dose used in in vitro assays. This resulted in a total concentration of 0.5 × 10^−7^, 1 × 10^−7^, and 1.5 × 10^−7^ M. The drug combinations were doxorubicin plus docetaxel, doxorubicin plus bicalutamide, docetaxel plus bicalutamide, and doxorubicin plus docetaxel plus bicalutamide. Untreated cells were used as control. Cells were cultured for a period of 24 h at a temperature of 37 °C in a humidified 5% CO_2_ atmosphere. On the following day, MTT was added, and the absorbance was measured at a wavelength of 568 nm with an automated plate reader (ThermoFisher Scientific, Waltham, MA, USA). Finally, data were expressed as a percentage of cell viability with respect to the control cells. The CI values were obtained using the unified theory introduced by Chou and Talalay based on the mass-action law [41], with the CompuSyn software 2004 version employed for this purpose (CompuSyn, Inc., New York, NY, USA). The CI is a quantitative representation of pharmacological interactions. Roughly, CI < 1 indicates synergism, CI = 1 indicates additive interaction, and CI > 1 indicates antagonism (Table 1).

### 4.7. Wound Healing Assay

IPC-366 and SUM149 were seeded in 24-well polystyrene plates (Corning Incorporated, New York, NY, USA) at a density of 10^5^ cells per well. Once the cells had reached a confluence of 90%, a wound was performed in the middle of the well, and the drugs were added at a concentration of 100 nM. Cells were cultured in a humified 5% CO2 atmosphere at 37 °C for 24 h. Images were captured for each well at the time the wound was performed and at the end of the assay using phase-contrast microscopy (Optika XDS-2 Inverted Microscope, Euromicroscopes, S.L., Barcelona, Spain). Culture media was collected at 24 h for hormonal analysis.

Results were processed using ImageJ MRI-Wound Healing Tool software 1.53e version, comparing the wound width at zero and 24 h for the different treatments with respect to the control. The data were obtained in pixels and represented as a percentage of wound closure with respect to the control.

### 4.8. Experimental Animals and Treatments

A total of 50 female Balb/SCID mice were obtained from Janvier Labs (Madrid, Spain) in the early morning, with dams to minimize shipping stress. The mice were then acclimatized for 7 days in the Animal Facility (Department of Animal Physiology, School of Veterinary Medicine, University Complutense of Madrid). The mice were housed in polycarbonate cages, with three mice per cage. The room was maintained at controlled environmental conditions (temperature: 23 ± 2 °C; relative humidity: 50 ± 10%; 10–15 air changes per hour; and a light:dark cycle of 12:12 h). Soy-free pellet food (Dyets, Inc., Bethlehem, Pennsylvania, USA) and water, previously sterilized, were provided ad libitum. The sample size needed to be high enough to compare the normal means of the five experimental groups simultaneously. No exclusion criteria were used. It was determined using the sample size determination module of the statistical package Statgraphics Centurion XVI version (Statpoint Technologies, Inc., Warrenton, VA, USA), resulting in a total of five mice per group studied. The Institutional Animal Care and Use Committee of the University Complutense of Madrid, Spain, approved the experimental protocols for this study (number: Proex 176/19). All procedures were performed in accordance with the Guide for the Care and Use of Laboratory Animals.

A suspension of 10^6^ IPC-366 cells or 3 × 10^6^ SUM149 cells diluted in PBS was inoculated in the mammary fat pad of 6-8-week-old female Balb/SCID mice with a 21-gauge syringe. The mice were inspected twice weekly for the development of tumors until a volume of 0.5 cm^3^ was reached. Then, mice were divided into five experimental groups per cell line: a control group (n = 5 mice per cell line) injected with saline solution, and four experimental groups (n = 5 mice per group and cell line) treated with bicalutamide isolated; doxorubicin and docetaxel; docetaxel and bicalutamide; and doxorubicin, docetaxel and bicalutamide, as was described in Section 2.2. Tumors were measured every other day with a caliper, and tumor volume was calculated by volume = ((length) × (width)^2^)/2 [62].

Prior to all procedures, animals were anesthetized with isoflurane (IsoVet) at 4% for induction and 1.5% for the maintenance of sedation. The fresh gas-flow rate was set at 0.5 L of oxygen per minute. At the end of treatment, animals were sacrificed by a lethal dose of isoflurane. For hormonal assays, tumors were excised and homogenized in PBS, and blood samples were collected with intracardiac puncture. The serum was extracted by centrifuging the samples for 20 min at 3600 rpm and 4 °C for subsequent hormonal analysis.

### 4.9. Steroid Determinations in Culture Media, Serum and Tumor Homogenates

Progesterone (P4), androstenedione (A4), estrone sulphate (E1SO4), 17β-estradiol (E2), and testosterone (T) antibodies were developed in the Department of Physiology (UCM, Spain). A previously validated competitive enzyme-immunoassay (EIA) was performed [9] to determine the hormone concentration in culture medium and tumor-homogenate samples, as well as a validated amplified EIA [63] for serum samples. Dihydroepiandrostenedione (DHEA) and dihydrotestosterone (DHT) determinations were performed using a commercially available EIA kit (DEH3344 and DE5761, respectively) (Demeditec, Germany) according to the manufacturer’s instructions. The hormones determined and the antibodies used are summarized in Table 2.

Hormone concentrations were calculated using software developed for this technique (ELISA AIS, Eurogenetics, Belgium). All hormone concentrations were expressed in ng/mL for culture medium and serum samples and in ng/g for tumor homogenates, except for DHT culture media, serum, and tumor-homogenate hormone concentrations, which were expressed in pg/mL and pg/g.

### 4.10. Statistics

Data were analyzed using IBM SPSS Statistics 28.0 software. A Shapiro-Wilk test was used to assess the goodness-of-fit distribution of the data. Since data were normally distributed, a one-way analysis of variance (ANOVA) was performed. After denoting that there was homogeneity of variances, a Bonferroni test was used to establish significant differences among experimental groups and control in sensitivity assay, cell viability, and wound-healing assays. For samples that were not normally distributed, a non-parametric Mann-Whitney U test was applied to search for significant differences between controls and treatments in tumor growth, serum, tumor-homogenate, and culture media hormone determinations, as well as mean fluorescence intensity. In all statistical comparisons, a *p* value < 0.05 was considered statistically significant.

## 5. Conclusions

The application of docetaxel as a single agent resulted in a reduction in cell proliferation in vitro in IPC-366 and SUM149 cells, but its combination with bicalutamide produced an additive effect, enhancing the reduction in cell migration in both cell lines. Therefore, docetaxel in IMC could be of interest to the clinic in the future due to it having been demonstrated to have good results in reducing tumor volume. The weak effect of doxorubicin as a single agent, in addition to the moderate antagonism effect of doxorubicin plus docetaxel in IPC-366, could be the reason why combination chemotherapy has not provided the expected efficacy in IMC. On the other hand, the addition of bicalutamide to doxorubicin plus docetaxel resulted in enhancing the treatment efficacy in IMC and IBC xenotransplanted mice. Therefore, the addition of bicalutamide to the combination of doxorubicin and docetaxel could be a potential therapeutic strategy in IMC and IBC treatment.

## Figures and Tables

**Table 1:** Range of CI and description of drug combination effect described by Chou and Talalay [41].

Range of Combination Index (CI)	Description
<0.1	Very strong synergism
0.1–0.3	Strong synergism
0.3–0.7	Synergism
0.7–0.85	Moderate synergism
0.85–0.90	Slight synergism
0.90–1.10	Nearly additive
1.10–1.20	Slight antagonism
1.20–1.45	Moderate antagonism
1.45–3.3	Antagonism
3.3–10	Strong antagonism
>10	Very strong antagonism

**Table 2:** Steroid hormones assayed and antibodies used for EIA determinations. DHEA and DHT were determined using commercial kits following manufacturer’s instructions.

Hormone	Abbreviation	Antibody Code	Dilution
Progesterone	P4	CL425	1/60,000
Estrone sulphate	E1SO4	R522-2	1/12,000
17β-estradiol	E2	C6-E91	1/4000
Testosterone	T	R156	1/8000
Androstenedione	A4	C9111	1/5000
Dihydroepiandrostenedione	DHEA	DE5761	
Dihydrotestosterone	DHT	DEH3344	

## Data Availability

Data are contained within the article.
